# Supplementary material for: A fault-intrusion-tolerant system and deadline-aware algorithm for scheduling scientific workflow in the cloud
Source: PeerJ Comput Sci. 2021 Nov 2;7:e747. doi: 10.7717/peerj-cs.747 (PMC8576554; doi:10.7717/peerj-cs.747)
Supplement: Supplemental Information 6 [file peerj-cs-07-747-s006.zip › Myproject1.1/docs/index-files/index-11.html]

L-Index


JavaScript is disabled on your browser.


- Overview
- Package
- Class
- Use
- Tree
- Deprecated
- Index
- Help

- Prev Letter
- Next Letter

- Frames
- No Frames

- All Classes

A B C D E F G H I J L M N O P Q R S T U V W Z 


## L

latency - Variable in class org.cloudbus.cloudsim.network.datacenter.Switch


length - Variable in class org.workflowsim.failure.FailureRecord
:   Length

level - Variable in class org.cloudbus.cloudsim.network.datacenter.Switch


linkDC - Static variable in class org.cloudbus.cloudsim.network.datacenter.NetDatacenterBroker


listToArray(List<? extends Number>) - Static method in class org.cloudbus.cloudsim.util.MathUtil
:   List to array.

loadHistory(String) - Method in class org.cloudbus.cloudsim.UtilizationModelStochastic
:   Load history.

Log - Class in org.cloudbus.cloudsim
:   The Log class used for performing loggin of the simulation process.

Log() - Constructor for class org.cloudbus.cloudsim.Log


logflag - Static variable in class org.cloudbus.cloudsim.network.datacenter.NetworkConstants


LognormalDistr - Class in org.cloudbus.cloudsim.distributions
:   The Class LognormalDistr.

LognormalDistr(Random, double, double) - Constructor for class org.cloudbus.cloudsim.distributions.LognormalDistr
:   Instantiates a new lognormal distr.

LognormalDistr(double, double) - Constructor for class org.cloudbus.cloudsim.distributions.LognormalDistr
:   Instantiates a new lognormal distr.

LomaxDistribution - Class in org.cloudbus.cloudsim.distributions
:   The Class LomaxDistribution.

LomaxDistribution(double, double, double) - Constructor for class org.cloudbus.cloudsim.distributions.LomaxDistribution
:   Instantiates a new lomax distribution.

LomaxDistribution(Random, double, double, double) - Constructor for class org.cloudbus.cloudsim.distributions.LomaxDistribution
:   Instantiates a new lomax distribution.

LrMc - Class in org.cloudbus.cloudsim.examples.power.planetlab
:   A simulation of a heterogeneous power aware data center that applies the Local Regression (LR) VM
    allocation policy and Maximum Correlation (MC) VM selection policy.

LrMc() - Constructor for class org.cloudbus.cloudsim.examples.power.planetlab.LrMc


LrMc - Class in org.cloudbus.cloudsim.examples.power.random
:   A simulation of a heterogeneous power aware data center that applies the Local Regression (LR) VM
    allocation policy and Maximum Correlation (MC) VM selection policy.

LrMc() - Constructor for class org.cloudbus.cloudsim.examples.power.random.LrMc


LrMmt - Class in org.cloudbus.cloudsim.examples.power.planetlab
:   A simulation of a heterogeneous power aware data center that applies the Local Regression (LR) VM
    allocation policy and Minimum Migration Time (MMT) VM selection policy.

LrMmt() - Constructor for class org.cloudbus.cloudsim.examples.power.planetlab.LrMmt


LrMmt - Class in org.cloudbus.cloudsim.examples.power.random
:   A simulation of a heterogeneous power aware data center that applies the Local Regression (LR) VM
    allocation policy and Minimum Migration Time (MMT) VM selection policy.

LrMmt() - Constructor for class org.cloudbus.cloudsim.examples.power.random.LrMmt


LrMu - Class in org.cloudbus.cloudsim.examples.power.planetlab
:   A simulation of a heterogeneous power aware data center that applies the Local Regression (LR) VM
    allocation policy and Minimum Utilization (MU) VM selection policy.

LrMu() - Constructor for class org.cloudbus.cloudsim.examples.power.planetlab.LrMu


LrMu - Class in org.cloudbus.cloudsim.examples.power.random
:   A simulation of a heterogeneous power aware data center that applies the Local Regression (LR) VM
    allocation policy and Minimum Utilization (MU) VM selection policy.

LrMu() - Constructor for class org.cloudbus.cloudsim.examples.power.random.LrMu


LrrMc - Class in org.cloudbus.cloudsim.examples.power.planetlab
:   A simulation of a heterogeneous power aware data center that applies the Local Regression Robust
    (LRR) VM allocation policy and Maximum Correlation (MC) VM selection policy.

LrrMc() - Constructor for class org.cloudbus.cloudsim.examples.power.planetlab.LrrMc


LrrMc - Class in org.cloudbus.cloudsim.examples.power.random
:   A simulation of a heterogeneous power aware data center that applies the Local Regression Robust
    (LRR) VM allocation policy and Maximum Correlation (MC) VM selection policy.

LrrMc() - Constructor for class org.cloudbus.cloudsim.examples.power.random.LrrMc


LrrMmt - Class in org.cloudbus.cloudsim.examples.power.planetlab
:   A simulation of a heterogeneous power aware data center that applies the Local Regression Robust
    (LRR) VM allocation policy and Minimum Migration Time (MMT) VM selection policy.

LrrMmt() - Constructor for class org.cloudbus.cloudsim.examples.power.planetlab.LrrMmt


LrrMmt - Class in org.cloudbus.cloudsim.examples.power.random
:   A simulation of a heterogeneous power aware data center that applies the Local Regression Robust
    (LRR) VM allocation policy and Minimum Migration Time (MMT) VM selection policy.

LrrMmt() - Constructor for class org.cloudbus.cloudsim.examples.power.random.LrrMmt


LrrMu - Class in org.cloudbus.cloudsim.examples.power.planetlab
:   A simulation of a heterogeneous power aware data center that applies the Local Regression Robust
    (LRR) VM allocation policy and Minimum Utilization (MU) VM selection policy.

LrrMu() - Constructor for class org.cloudbus.cloudsim.examples.power.planetlab.LrrMu


LrrMu - Class in org.cloudbus.cloudsim.examples.power.random
:   A simulation of a heterogeneous power aware data center that applies the Local Regression Robust
    (LRR) VM allocation policy and Minimum Utilization (MU) VM selection policy.

LrrMu() - Constructor for class org.cloudbus.cloudsim.examples.power.random.LrrMu


LrrRs - Class in org.cloudbus.cloudsim.examples.power.planetlab
:   A simulation of a heterogeneous power aware data center that applies the Local Regression Robust
    (LRR) VM allocation policy and Random Selection (RS) VM selection policy.

LrrRs() - Constructor for class org.cloudbus.cloudsim.examples.power.planetlab.LrrRs


LrrRs - Class in org.cloudbus.cloudsim.examples.power.random
:   A simulation of a heterogeneous power aware data center that applies the Local Regression Robust
    (LRR) VM allocation policy and Random Selection (RS) VM selection policy.

LrrRs() - Constructor for class org.cloudbus.cloudsim.examples.power.random.LrrRs


LrRs - Class in org.cloudbus.cloudsim.examples.power.planetlab
:   A simulation of a heterogeneous power aware data center that applies the Local Regression (LR) VM
    allocation policy and Random Selection (RS) VM selection policy.

LrRs() - Constructor for class org.cloudbus.cloudsim.examples.power.planetlab.LrRs


LrRs - Class in org.cloudbus.cloudsim.examples.power.random
:   A simulation of a heterogeneous power aware data center that applies the Local Regression (LR) VM
    allocation policy and Random Selection (RS) VM selection policy.

LrRs() - Constructor for class org.cloudbus.cloudsim.examples.power.random.LrRs

A B C D E F G H I J L M N O P Q R S T U V W Z

- Overview
- Package
- Class
- Use
- Tree
- Deprecated
- Index
- Help

- Prev Letter
- Next Letter

- Frames
- No Frames

- All Classes
